# Integrated Psychological Care of an Adult With Congenital Heart Disease

**DOI:** 10.1016/j.jaccas.2025.106206

**Published:** 2026-03-13

**Authors:** Michael T.M. Finn, Gruschen R. Veldtman

**Affiliations:** a Betz Congenital Heart Center, Helen DeVos Children’s Hospital Corewell Health, Grand Rapids, Michigan, USA; b Department of Pediatrics and Human Development, Michigan State University College of Human Medicine, Grand Rapids, Michigan, USA

**Keywords:** congenital heart disease, postsurgical, psychotherapy, treatment

## Abstract

**Background:**

Congenital heart disease (CHD) affects 1% of live births globally. Although depression and anxiety are commonly recognized in adults with CHD, psychological challenges extend beyond traditional diagnoses to include regulatory flexibility and cognitive adaptability, which influence coping strategies.

**Case Summary:**

A 40-year-old woman with a history of complex CHD presented for psychological support before major cardiac surgery. The patient described feeling “at war” with her body, demonstrating patterns of counterdependency and analytical problem-solving. Assessment revealed specific deficits in cognitive flexibility and mentalization capacity. Psychodynamic treatment spanning 56 sessions across presurgical, surgical, and postsurgical phases addressed these regulatory patterns. By treatment conclusion, PROMIS (Patient-Reported Outcomes Measurement Information System) Global Health scale showed substantial improvements in both physical health (T score increased from 16 to 39.8) and mental health (T score increased from 31.3 to 41.1) domains. The patient reported a shift from feeling “at war” to being “at home” in her body by the end of the treatment.

**Discussion:**

Regulatory flexibility provides a valuable framework for understanding psychological adaptation in CHD patients. By honoring existing strengths while addressing rigid coping styles, psychotherapy can help adults with CHD develop more sustainable adaptation patterns for both physical and psychological well-being.

## Visual Summary

**Figure:**
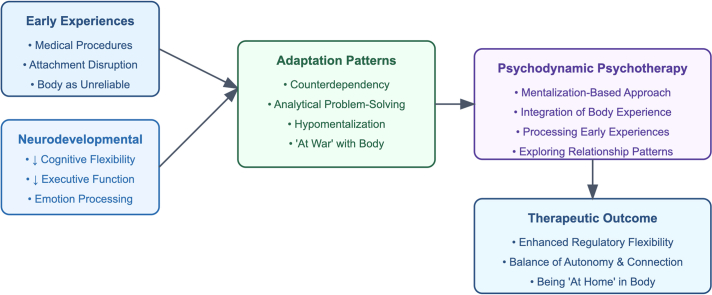

**Visual Summary:**
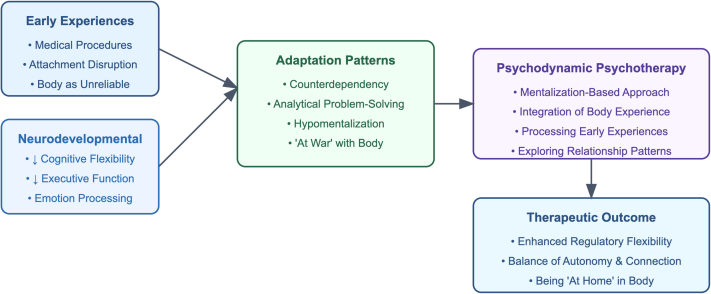
Conceptual Diagram of the Diagnostic Approach and Psychotherapy Process

Introduction Congenital heart disease (CHD), affecting 1% of live births globally, requires ongoing medical care because of significant risks of complications.^1^^,^^2^ Although depression and anxiety are commonly recognized concerns in adult CHD (ACHD), psychological challenges extend beyond traditional psychiatric diagnoses. The phenomenological experience of suffering in chronic illness has been described as an “unhomelike” being in the world, a profound disruption in one’s situation in the world, particularly their body.^3^ Recent research has highlighted how early medical experiences in CHD can create specific neurodevelopmental vulnerabilities in executive functioning and cognitive flexibility that persist into adulthood, shaping both personality development and adaptation.^4^ In fact, recent research has, for the first time, revealed high rates of personality dysfunction in ACHD, in part related to childhood adversity (eg, neglect), which tend toward struggles with rigid control.^5^ Evidence points toward regulatory flexibility as a key transdiagnostic process supporting adaptive coping with stress and post-traumatic stress.^6^ Different approaches to psychotherapy, including psychodynamic psychotherapy,^7^ have the potential to support flexible self-regulation toward a more “homelike” experience.

**Take-Home Messages**

• Adults with CHD may develop rigid coping patterns rooted in early medical experiences and neurodevelopmental vulnerabilities associated with CHD that extend beyond conventional psychiatric diagnoses, requiring assessment of cognitive flexibility and mentalization capacity rather than depression and anxiety screening alone.
• Psychodynamic psychotherapy targeting regulatory flexibility can facilitate meaningful shifts in how patients with CHD relate to their bodies and navigate medical challenges, transforming feelings of being “at war” with physical limitations into a more integrated adaptive relationship with chronic illness.

## Case Presentation

The patient’s experience of living with complex CHD, in the form of ventricular septal defect with infundibular stenosis and anomalous origin of the left coronary artery from the right sinus with proximal intramural course, illustrates how both early experiences and vulnerabilities can profoundly shape patterns of adaptation. Required to undergo her first heart surgery at 5 months old for ventricular septal defect, the patient grew up in an environment where her body was experienced as fundamentally unreliable. Her mother’s response—referring to her as “the broken one” and expressing when no one else was around that “it would have been easier if you didn’t live”—perhaps most directly reinforced this feeling of precariousness and unsafety.

The patient’s identity was deeply rooted in the ranching culture of the western United States, where she had previously managed a ranch with many large animals. This cultural context emphasized values of physical endurance and independence, which aligned with her values and personality but sometimes came at a significant cost. Her pattern of counterdependency (actively minimizing her needs for care and support) reflected remarkable independence but posed significant barriers to optimal self-regulation.

When the patient presented for integrated psychological care by referral from congenital cardiology, she was 40 years old and facing another major cardiac surgery for myocardial ischemia due to the proximal intramural obstruction and the slit-like origin of her anomalous left coronary artery. She described feeling “trapped” and “at war” with her “deteriorating” body. Although certainly coping with congenital conditions, it was observed that many of her symptoms were not fully explained by known defects. Her relational history included being abandoned during an inpatient hospital stay in another state by her ex-husband, who at other times left her alone for days on end to care for many large animals on their ranch. The patient would often push herself well beyond a comfortable physical capacity to meet the daily needs of her animals and continued that same work ethic in her daily life. However, symptoms would often pose a substantial and frustrating barrier to carrying out basic tasks around the house.

Initial psychological assessment revealed that despite being negative on brief screeners of anxiety and depression, she showed persistent patterns of cognitive rigidity and perseverative thinking. This can be understood from a psychodynamic framework as a retreat from emotional processing into concrete physical experience, where bodily symptoms become the primary language of distress. The patient demonstrated particular difficulty integrating emotional and bodily experiences, instead relying heavily on concrete, analytical problem-solving that often became unproductive and self-critical. Hypomentalization has an alienating and isolating quality, which patterns neatly with rigid self-reliance, also called *counter*dependency. We collaboratively identified this as an inflexible and context-insensitive way of coping.^7^ These dynamics qualitatively aligned with the emerging emphasis on cognitive flexibility as a core vulnerability in CHD and a place of significant opportunity for intervention.^4^

Treatment spanned approximately 900 days, comprising 56 sessions across presurgical, surgical, and postsurgical phases, with open heart surgery occurring around session 32 and some sessions conducted during inpatient recovery. The majority of sessions (52) were conducted via telehealth because of the patient’s significant driving distance from the medical center in a rural area which was 1 of only 2 congenital heart centers serving the state. Drawing on mentalization-based principles within a psychodynamic framework,^6^ our work focused on helping the patient develop greater understanding of patterns of relating with herself and others and implement more adaptive and flexible coping.

During her hospital stay for open heart surgery for unroofing of a myocardial bridge and left coronary ostioplasty, an important moment of coping occurred when the patient’s mother left the room during a moment of significant distress, saying, “I can’t see this.” What previously may have been an incredibly distressing experience, the patient was able to mentalize her emotional response and implement an adaptive and flexible coping response by requesting space and time from her mother (“I decide”) while seeking support from her partner.

As her physical well-being improved postsurgery, the patient began transforming her relationship with both her body and her developed patterns of adaptation. Alongside cardiac rehabilitation experience, we further worked to enhance the flexible “art” of listening to and responding compassionately to bodily signals as she was starting to experience some shifting limits. She embraced a mentorship role at a local ranch, teaching horsemanship to a young girl, and over time became a cherished member of the community. She also made major changes in close relationships. The ranching experience had changed profoundly: where it was once a place of pushing beyond limits, it was now a place of community, connection, and flexible coping.

**Figure 1:**
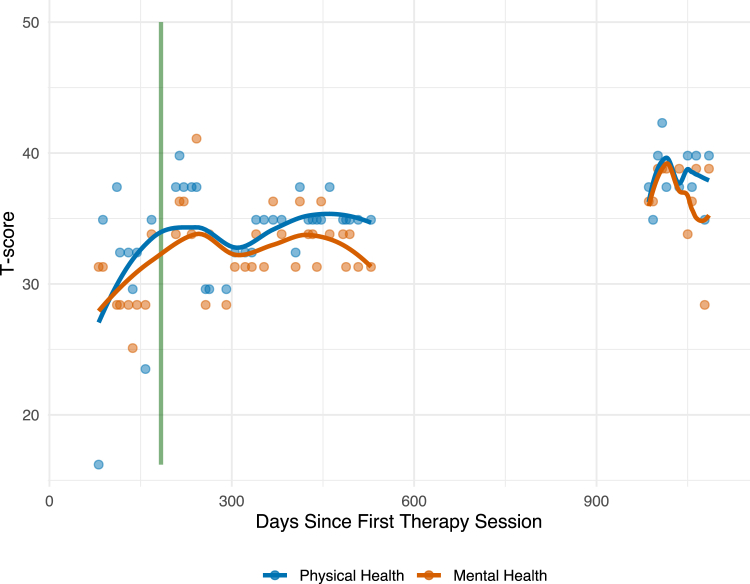
PROMIS Global Health Outcomes Over Time Administered at Each Psychotherapy Session PROMIS = Patient-Reported Outcomes Measurement Information System.

The PROMIS (Patient-Reported Outcomes Measurement Information System) Global Health scale documented gradual improvement in both physical and mental health domains (Figure 1). The patient’s Physical Health T score increased from 16 to 39.8, whereas her Mental Health T score increased from 31.3 to 41.1. In one of the final sessions during this time frame, she described a general sense of having built a new way of being “at home” in her body and world, which had changed much in quality from the daily “at war” experience at the outset of our work.

## Discussion

This case illustrates how vulnerabilities and early developmental experiences related to CHD may shape an unhomelike way of relating to oneself,^3^ which reflects in personality development and patterns of adaptation. CHD can increase risk for specific vulnerabilities in cognitive flexibility that persist into adulthood.^4^ We describe alignment across psychodynamic approaches,^6^ an emerging understanding of cognitive flexibility in CHD across the lifespan,^4^ and emerging research on personality functioning in ACHD,^5^ suggesting how psychotherapy can support the development and implementation of new possibilities of coping. Although psychodynamic psychotherapy is particularly well-suited for treating personality functioning and entrenched styles of coping,^6^ theses ideas can be integrated into other theoretical orientations for psychotherapy.^7^ Continued work in this area may enhance the efficiency and quality of psychological interventions for adults with CHD.

## Perspectives

Reading this did move me a bit and stir up a few emotions, naturally as they are my memories. Thank you for the delicate interpretation, compassion and wording. Your continued help and guidance truly continues to make a difference in my life.

## Funding Support and Author Disclosures

The authors have reported that they have no relationships relevant to the contents of this paper to disclose.
